# High tumor mutation burden indicates better prognosis in colorectal cancer patients with *KRAS* mutations

**DOI:** 10.3389/fonc.2022.1015308

**Published:** 2022-11-14

**Authors:** Jianlei Wang, Jianping Song, Zeyang Liu, Tingxiao Zhang, Yanfeng Liu

**Affiliations:** ^1^ Department of Organ Transplantation, Qilu Hospital, Cheeloo College of Medicine, Shandong University, Jinan, Shandong, China; ^2^ Department of General Surgery, The Second Hospital of Shandong University, Jinan, Shandong, China; ^3^ Department of General Surgery, Qilu Hospital, Cheeloo College of Medicine, Shandong University, Jinan, Shandong, China

**Keywords:** tumor mutation burden, colorectal cancer, *KRAS* mutation, prognosis, nomogram

## Abstract

**Objective:**

Colorectal cancer (CRC) is a common type of malignant tumor of the digestive tract. Tumor mutation burden (TMB) is a potential prognostic indicator of numerous malignant tumors. This study investigated the prognostic value of TMB in CRC.

**Methods:**

This study analyzed the clinical and somatic mutation data of patients with CRC from the Memorial Sloan Kettering Cancer Center (MSKCC) and The Cancer Genome Atlas (TCGA) cohorts. The genetic landscape was visualized using the maftools package in R software. Survival curves were constructed using the Kaplan–Meier method, and Cox regression analysis was performed to confirm that TMB is an independent prognostic indicator. A nomogram was developed to construct the prognostic model, which was evaluated using the C-index, calibration curve, and decision curve analysis.

**Results:**

In patients with CRC, *APC* mutations indicated longer overall survival (OS), whereas *KRAS* mutations indicated shorter OS. For all included patients, there was no significant difference in the OS between the TMB-high and TMB-low groups. For patients with *KRAS* mutations, the OS in the TMB-high group was longer than that in the TMB-low group. Cox regression analysis showed that TMB was an independent prognostic factor in CRC patients with *KRAS* mutations. This explains the good accuracy of the nomogram prognostic model using TMB and indicates its good prospect in clinical applications.

**Conclusions:**

A high TMB indicates better prognosis in CRC patients with *KRAS* mutations, thus confirming the value of TMB in clinical applications.

## Introduction

Colorectal cancer (CRC) is the third most prevalent malignant tumor worldwide, with high morbidity and mortality (1). Although scientific and clinical advances in its early detection and surgery have increased the 5-year survival rates to 90% and 71% for localized and regionalized CRC, respectively, the 5-year survival rate for metastatic CRC remains low at 14% (2). Routine treatments for CRC currently include surgery, radiotherapy, and chemotherapy (3). In patients with advanced tumors, radiotherapy and chemotherapy elicit numerous side effects owing to their low specificity and their cytotoxicity toward growing and dividing cells (4). Cancer immunotherapy, which involves harnessing the immune system to attack the cancer, is a promising approach to the treatment of CRC (2). However, many patients nonetheless have poor therapeutic outcomes during clinical treatment. There is, therefore, a clear need to identify predictive biomarkers to guide CRC treatment.

Tumor mutation burden (TMB) is defined as the total number of somatic coding errors, base substitutions, and indel mutations found per million bases of DNA, and it can effectively estimate both the mutational and neoantigen loads (5, 6). TMB was first identified as a latent biomarker for immune checkpoint inhibitors (ICIs) in melanoma (7), and it has since been suggested that a high TMB is related to the effectiveness of ICIs. There are indeed indications that TMB could more broadly be a useful indicator of the efficiency of immunotherapy (8, 9). Additionally, TMB is a potential prognostic indicator, although the relationship between TMB and the prognosis of patients with tumors is still a matter of debate. Ballman et al. (10) and Riviere et al. (11) reported that a high TMB was intimately associated with longer survival. Samstein et al. (12) reported no correlation between a high TMB and a more favorable prognosis in patients with advanced cancers who were not treated with ICIs. However, a high TMB has been reported to indicate a worse prognosis for thyroid carcinoma (13), head and neck squamous cell carcinoma (14), and intrahepatic cholangiocarcinoma (15). There have only been a few reports to date on the correlation between TMB and the survival of patients with CRC.

In this study, we analyzed the somatic mutation data and clinical characteristics of patients with CRC in the Memorial Sloan Kettering Cancer Center (MSKCC) and The Cancer Genome Atlas (TCGA) cohorts and determined the most common mutations in driver genes in CRC. The association between TMB and the prognosis of patients with CRC was then explored in terms of the different driver gene mutations, and it was found that TMB is an independent prognostic factor for CRC patients with *KRAS* mutations.

## Materials and methods

### Data collection and processing

The clinical information and somatic mutation data of 1,099 patients with CRC from the MSKCC cohort (16) (https://www.cbioportal.org/study/summary?id=crc_msk_2017) and 556 patients with CRC from TCGA cohort (https://portal.gdc.cancer.gov/) were included. All included data were from patients with CRC confirmed by biopsy or operation resection. If the MSKCC database contained data from multiple samples from the same patient, only the most recent data were used. Data with missing clinical information were excluded from the corresponding analyses. TMB was defined as the number of somatic, non-silent, protein-coding mutations in the coding regions per megabase (mut/Mb). The genetic landscape was visualized using the maftools package in R software. Ethics approval was not required for the study. The datasets used and analyzed in this study are publicly available, and the clinicopathological information, including age, sex, TMB, primary tumor location, and stage at diagnosis, was reviewed retrospectively.

### Survivability and Cox regression analysis

Based on a TMB cutoff of 10 mut/Mb (17, 18), a total of 1,376 patients with TMB ≤ 10 mut/Mb were assigned into the TMB-low category, while 278 patients with TMB > 10 mut/Mb (one patient lacked TMB data) were included into the TMB-high category. Survival curves were constructed according to the Kaplan–Meier method, and differences between the curves were examined using a log-rank test. A *p*-value <0.05 was considered statistically significant. Cox regression analysis was then performed to predict whether TMB could be used as an independent prognostic indicator for CRC (674 CRC patients with *KRAS* mutations and complete clinical information were included).

### Construction and validation of the nomogram

To maximize the statistical power and minimize bias in the analysis, propensity score matching was performed with a 7:3 ratio. The following covariates that might affect the prognostic outcomes were included: sex, age, primary tumor location, stage at diagnosis, and TMB. The training and validation groups were analyzed using the chi-square test. The predictive accuracy and the discrimination of the nomogram were assessed using the concordance index (C-index) and decision curve analysis (DCA). A calibration curve was used to compare the degree of agreement between the predicted probabilities of the nomogram and the actual observations.

### Statistical analysis

Statistical analyses were performed using IBM Corporation SPSS version 25.0 software. The survival, rms, and stdca.R packages in R (version 4.1.0) were used for the analysis of the prognostic model, and the curves were drawn using the ggplot2 package in R. Statistical significance was set at *p* < 0.05 in our analyses.

## Results

### Cohort characteristics

In this study, the MSKCC and TCGA cohorts included 1,099 and 556 patients with CRC, respectively. We used the 341- (IMPACT341) and 410-gene (IMPACT410) panels for the majority of the patients in the MSKCC cohort. Compared to the most recent 468-gene panel (IMPACT468), the unsequenced genes in the earlier versions were assumed to be wild type or non-mutated. Patients in TCGA cohort were examined using whole-exome sequencing (WES), which is considered the gold standard for the calculation of TMB values. The clinical data in this study included age (<60 and ≥60 years), sex (female/male), TMB [low (≤ 10 mut/Mb) and high (>10 mut/Mb)], primary tumor location (left or right), and stage at diagnosis (I–IV). The baseline clinicopathological features of the study cohort and the number of participants with missing data for each variable of interest are listed in **Table 1**.

**Table 1:** Clinical characteristics of the study population.

Characteristics	MSKCC patients (*n* = 1,099)		TCGA patients (*n* = 556)	
	n	%	n	%
Age (years)				
Median			67	
Range			31–90	
<60	701	63.8	167	30.0
≥60	398	36.2	389	70.0
Sex				
Female	502	45.7	265	47.7
Male	597	54.3	291	52.3
TMB				
Median	6.1		2.8	
Range	0.9–336.8		0.2–299.0	
Low (≤10 mut/Mb)	905	82.3	471	84.7
High (>10 mut/Mb)	194	17.7	85	15.3
Unknown	1	0.1	0	0.0
Primary tumor location				
Left	760		309	55.6
Right	325		193	34.7
Unknown	14		54	9.7
Stage at diagnosis				
I	39	3.5	99	17.8
II	129	11.7	202	36.3
III	267	24.3	160	28.8
IV	664	60.4	74	13.3
Unknown	0	0.0	21	3.8
Overall survival (months)				
Median	27.6		2.8	
Range	0–292.9		0–137.4	
Unknown	0	0.0	2	0.4

TMB, tumor mutation burden; MSKCC, Memorial Sloan Kettering Cancer Center; TCGA, The Cancer Genome Atlas.

### Landscape of genetic mutation profiles in CRC

**Figure 1:**
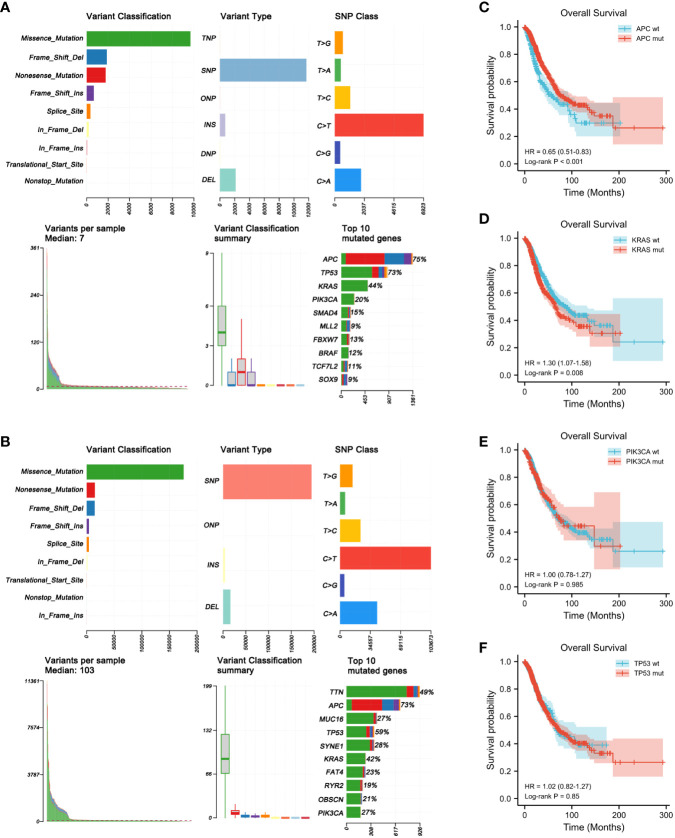
Genome-wide mutation profiling of patients with colorectal cancer (CRC). **(A, B)** Cohort summary plots for the Memorial Sloan Kettering Cancer Center (MSKCC) **(A)** and The Cancer Genome Atlas (TCGA) **(B)** cohorts displaying the distribution of variants according to variant classification, type, and single nucleotide variation (SNV) class. The *bottom panel* (from *left* to *right*) indicates the mutation load for each sample and variant classification type. A stacked bar plot shows the top 10 mutated genes. **(C–F)** Kaplan–Meier analysis showing the impact of gene mutations on the overall survival (OS) of patients with CRC (1,653 patients from the MSKCC and TCGA cohorts with complete OS data). **(C)** CRC patients with *APC* mutations had longer OS than those with wild-type *APC* (HR = 0.65, *p* < 0.001). **(D)** CRC patients with *KRAS* mutations had shorter OS than those with wild-type *KRAS* (HR = 1.30, *p* = 0.008). **(E, F)** *PIK3CA* (HR = 1.00, *p* = 0.985) **(E)** and *TP53* (HR = 1.02, *p* = 0.850) **(F)** mutations had no obvious effect on the OS of patients with CRC.

The gene mutation profiles of 1,099 and 556 patients with CRC (1,134 CRC samples) from the MSKCC and TCGA cohorts, respectively, were analyzed using maftools in R software (**Figures 1A, B** **)**. In brief, these mutations were further classified according to their categories, which were primarily missense mutations. Single nucleotide polymorphisms were more common than insertions or deletions. In terms of single nucleotide variations (SNVs), C>T was the most common in both the MSKCC and TCGA cohorts. The somatic mutation characteristics of the two cohorts were highly similar. The number of variants per sample in TCGA cohort was higher than that of the MSKCC cohort due to TCGA mutation data being examined using WES. The top 10 mutated genes in the MSKCC cohort were *APC* (75%), *TP53* (73%), *KRAS* (44%), *PIK3CA* (20%), *SMAD4* (15%), *MLL2* (9%), *FBXW7* (13%), *BRAF* (12%), *TCF7L2* (11%), and *SOX9* (9%) (**Figure 1A**), while the top 10 mutated genes in TCGA cohort were *TTN* (49%), *APC* (73%), *MUC16* (27%), *TP53* (59%), *SYNE1* (28%), *KRAS* (42%), *FAT4* (23%), *RYR2* (19%), *OBSCN* (21%), and *PIK3CA* (27%) (**Figure 1B**). Thus, both cohorts overlapped in terms of mutations of *APC*, *KRAS*, *PIK3CA*, and *TP53*.

### Prognostic value of the mutant genes

To determine the prognostic significance of the frequently mutated genes in CRC, we performed survival analysis using the Kaplan–Meier method with the log-rank test. We divided the patients into two groups (wild-type and mut-type) according to their gene mutation status (**Table 2**). Survival analysis indicated that CRC patients with *APC* mutations had longer overall survival (OS) than those with wild-type *APC* [hazard ratio (HR) = 0.65, *p* < 0.001] (**Figure 1C**). CRC patients with *KRAS* mutations had worse prognosis (HR = 1.30, *p* = 0.008) than those with wild-type *KRAS* (**Figure 1D**). *PIK3CA* and *TP53* mutations had no obvious effect on the prognosis of patients with CRC (*PIK3CA*: HR = 1.00, *p* = 0.985; *TP53*: HR = 1.02, *p* = 0.850) (**Figures 1E, F** **)**.

**Table 2:** Correlation between gene mutation and overall survival.

Gene	Median survival (months)		Log-rank test	
	Mutation	Wild type	HR (95% CI)	*p*-value
*APC*	75.8 (*n* = 1,238)	56.9 (*n* = 415)	0.65 (0.51–0.83)	<0.001
*KRAS*	64.7 (*n* = 720)	87.9 (*n* = 933)	1.30 (1.07–1.58)	0.008
*PIK3CA*	75.8 (*n* = 374)	70.7 (*n* = 1279)	1.00 (0.78–1.27)	0.985
*TP53*	72.5 (*n* = 1,130)	66.9 (*n* = 523)	1.02 (0.82–1.27)	0.850

APC, adenomatous polyposis coli; KRAS, Kirsten rat sarcoma viral oncogene homolog; PIK3CA, phosphatidylinositol-4,5-bisphosphate 3-kinase, catalytic subunit alpha; TP53, tumor protein p53; HR, hazard ratio.

### Prognostic impact of TMB

**Figure 2:**
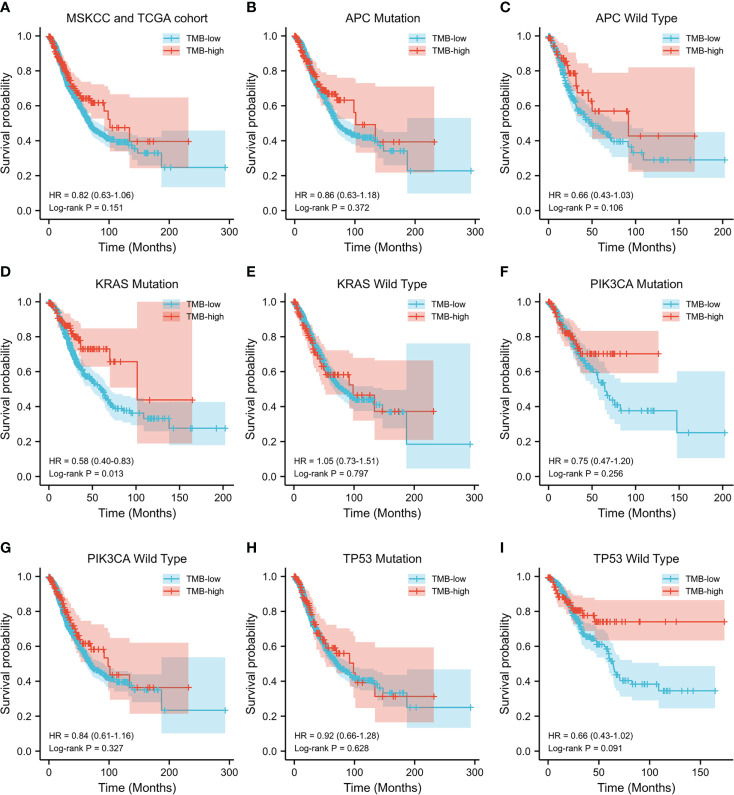
Evaluation of the tumor mutation burden (TMB) to predict the prognosis of patients with colorectal cancer (CRC). **(A)** Kaplan–Meier curve showing the impact of TMB on the overall survival (OS) of all CRC patients with complete TMB and OS data [1,652 patients from the Memorial Sloan Kettering Cancer Center (MSKCC) and The Cancer Genome Atlas (TCGA) cohorts] in this study (HR = 0.82, *p* = 0.151). **(B–I)** Kaplan–Meier analysis showing the impact of TMB on the OS of CRC patients with *APC* mutations (HR = 0.86, *p* = 0.372) **(B)**, wild-type *APC* (HR = 0.66, *p* = 0.106) **(C)**, *KRAS* mutations (HR = 0.58, *p* = 0.013) **(D)**, wild-type *KRAS* (HR = 1.05, *p* = 0.797) **(E)**, *PIK3CA* mutations (HR = 0.75, *p* = 0.256) **(F)**, wild-type *PIK3CA* (HR = 0.84, *p* = 0.327) **(G)**, *TP53* mutations (HR = 0.92, *p* = 0.628) **(H)**, and wild-type *TP53* (HR = 0.66, *p* = 0.091) **(I)**.

Subsequently, the prognostic value of TMB was investigated. The median TMB of the participants was 5.1 mut/Mb (range = 0.2–336.8 mut/Mb). Using a TMB cutoff of 10 mut/Mb, we stratified the patients into TMB-high (≥10 mut/Mb) and TMB-low (<10 mut/Mb). For the entire cohort of CRC patients with complete TMB and OS data in this study, no significant disparity in OS was observed between the TMB-high and TMB-low groups (HR = 0.82, *p* = 0.151) (**Figure 2A**). We then analyzed the survival predictive value of TMB in CRC patients with specific somatic mutation backgrounds. *APC* mutation did not affect the survival predictive value of TMB, as there was no obvious disparity in the OS data between the TMB-high and TMB-low groups (*APC* mutation: HR = 0.86, *p* = 0.372; wild-type *APC*: HR = 0.66, *p* = 0.106) (**Figures 2B, C** **)**. For patients with *KRAS* mutations, the OS was longer in the TMB-high group than that in the TMB-low group (HR = 0.58, *p* = 0.013) (**Figure 2D**), whereas there was no difference in the OS of patients with wild-type *KRAS* (HR = 1.05, *p* = 0.797) (**Figure 2E**). *PIK3CA* mutation did not affect the survival predictive value of TMB, as there was no significant difference in the OS data between the TMB-high and the TMB-low group (*PIK3CA* mutation: HR = 0.75, *p* = 0.256; *PIK3CA* wild type: HR = 0.84, *p* = 0.327) (**Figures 2F, G** **)**. In CRC patients with *TP53* mutations, a similar OS was observed in the TMB-high and TMB-low groups (HR = 0.92, *p* = 0.628) (**Figure 2H**). In patients with wild-type *TP53*, the TMB-high group tended to have longer OS than the TMB-low group (HR = 0.66, *p* = 0.091) (**Figure 2I**). Since the number of patients reaching the expected events in the TMB-high group was too small, the *p*-value obtained was not significant. Overall, a high TMB indicated better prognosis in CRC patients with *KRAS* mutations. The analysis result were listed in **Table 3**.

**Table 3:** Correlation between TMB and overall survival.

	Median survival (months)		Log-rank test	
	TMB-high	TMB-low	HR (95% CI)	*p*-value
All patients	101.4 (*n* = 278)	68.1 (*n* = 1374)	0.82 (0.63–1.06)	0.151
*APC* mutation	101.4 (*n* = 189)	70.7 (*n* = 1049)	0.86 (0.63–1.18)	0.372
*APC* wild type	91.8 (*n* = 89)	46.9 (*n* = 325)	0.66 (0.43–1.03)	0.106
*KRAS* mutation	101.4 (*n* = 126)	59.9 (*n* = 594)	0.58 (0.40–0.83)	0.013
*KRAS* wild type	98.4 (*n* = 152)	82.5 (*n* = 780)	1.05 (0.73–1.51)	0.797
*PIK3CA* mutation	– (*n* = 123)	64.7 (*n* = 251)	0.75 (0.47–1.20)	0.256
*PIK3CA* wild type	98.4 (*n* = 155)	69.1 (*n* = 1123)	0.84 (0.61–1.16)	0.327
*TP53* mutation	98.4 (*n* = 132)	71.9 (*n* = 998)	0.92 (0.66–1.28)	0.628
*TP53* wild type	– (*n* = 146)	63.9 (*n* = 376)	0.66 (0.41–1.07)	0.091

TMB, tumor mutation burden; APC, adenomatous polyposis coli; KRAS, Kirsten rat sarcoma viral oncogene homolog; PIK3CA, phosphatidylinositol-4,5-bisphosphate 3-kinase, catalytic subunit alpha; TP53, tumor protein p53; HR, hazard ratio.

### Cox regression analysis of CRC patients with *KRAS* mutations

To identify the independent markers with prognostic significance, we performed a multivariate Cox regression analysis to explore the correlation between OS and specific factors, including age, sex, primary tumor location, and stage at diagnosis, in CRC patients with *KRAS* mutations. In total, 674 patients with complete clinical information were included in the analysis. The results of the analysis suggested that TMB was an independent prognostic predictor for CRC patients with *KRAS* mutations (HR = 0.60, 95% CI = 0.38–0.94, *p* = 0.026). Additionally, age (<60 and ≥60 years) was also an independent prognostic factor for CRC patients with *KRAS* mutations (HR = 1.39, 95% CI = 1.03–1.87, *p* = 0.030) (**Table 4**).

**Table 4:** Univariate and multivariate analyses of overall survival.

	Univariate			Multivariate		
	HR	95% CI	*p*-value	HR	95% CI	*p*-value
Age (years)						
<60 (*n* = 336)	Ref			Ref		
≥60 (*n* = 338)	1.25	0.94–1.68	0.123	1.39	1.03–1.87	0.030
Sex						
Female (*n* = 330)	Ref			Ref		
Male (*n* = 344)	1.09	0.81–1.45	0.576	1.18	0.88–1.58	0.274
TMB						
Low (*n* = 555)	Ref			Ref		
High (*n* = 119)	0.57	0.40–0.82	0.012	0.60	0.38–0.94	0.026
Primary tumor location						
Left (*n* = 388)	Ref			Ref		
Right (*n* = 286)	1.13	0.84–1.51	0.417	1.17	0.87–1.57	0.294
Stage at diagnosis						
I (*n* = 48)	Ref			Ref		
II (*n* = 124)	1.11	0.34–3.65	0.867	0.97	0.28–3.34	0.959
III (*n* = 172)	1.84	0.74–4.58	0.299	2.00	0.62–6.49	0.245
IV (*n* = 330)	2.29	1.05–5.00	0.142	2.46	0.78–7.77	0.125

TMB, tumor mutation burden; HR, hazard ratio; CI, confidence interval.

### Construction of a prognostic model

**Figure 3:**
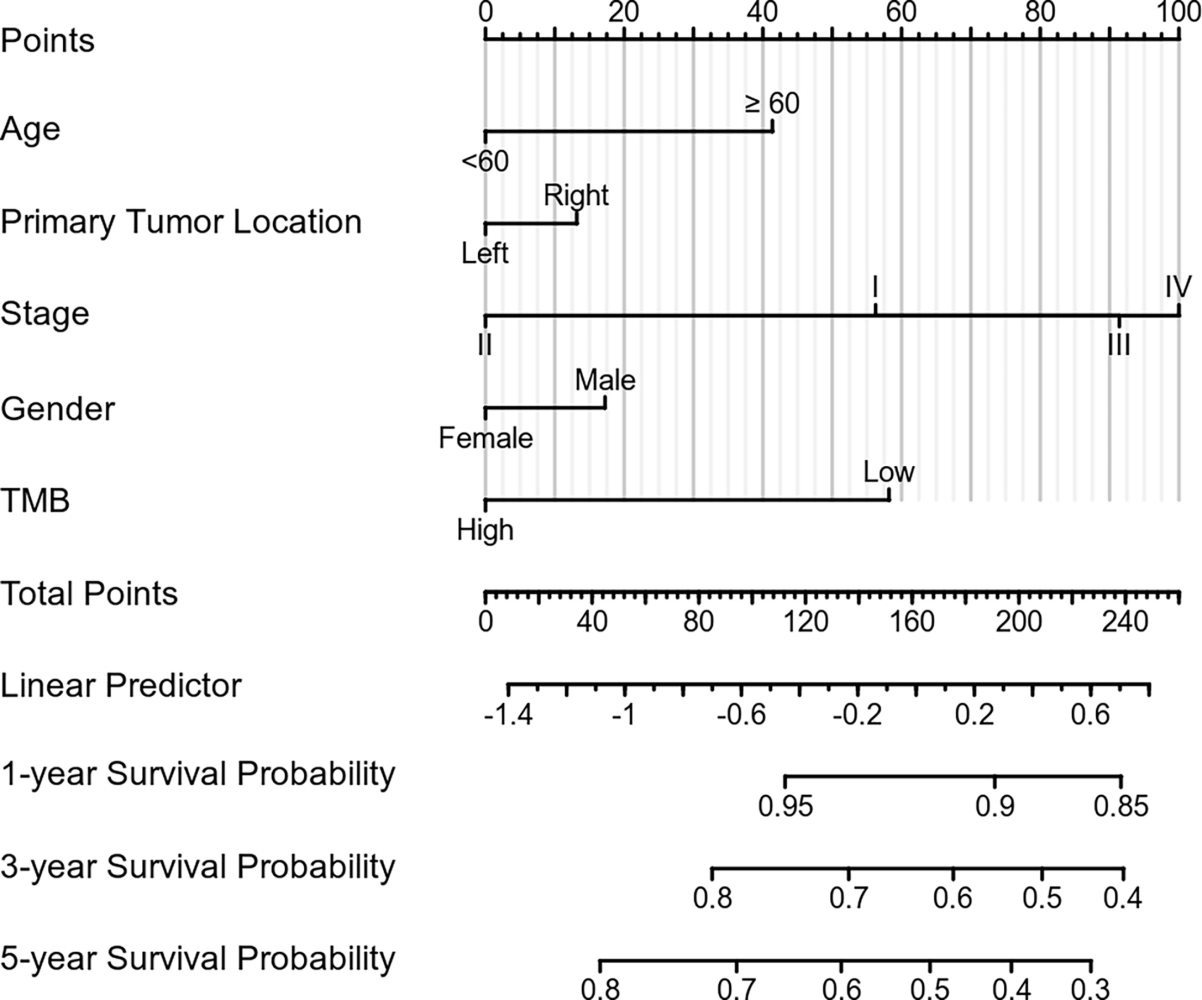
Construction of the prognostic nomogram. Predicted 1-, 3-, and 5-year survival rates in colorectal cancer (CRC) patients with *KRAS* mutations based on our nomogram, which included age, sex, tumor mutation burden, primary tumor location, and stage at diagnosis (C-index = 0.594, 95% CI = 0.566–0.622).

**Figure 4:**
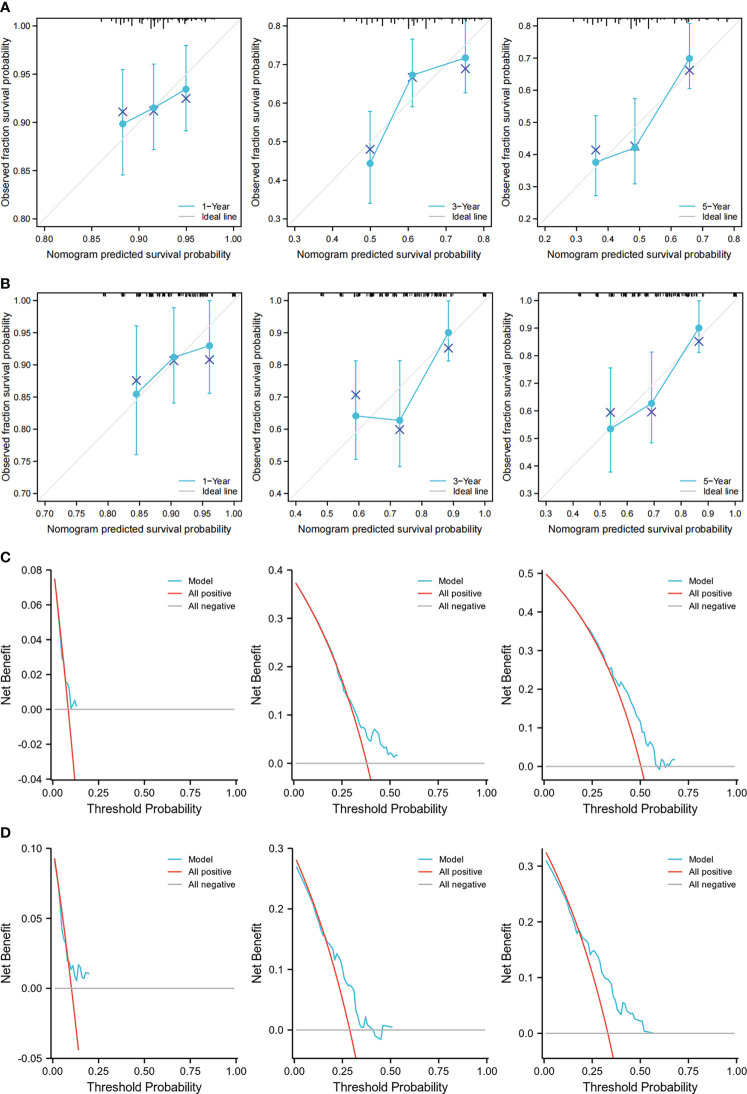
Calibration curves and decision curve analysis (DCA) of the nomogram. **(A, B)** Calibration curves showing the observation and prediction results of the 1-, 3-, and 5-year survival rates of patients in the training group (C-index = 0.594, 95% CI = 0.566–0.622) **(A)** and those in the validation group (C-index = 0.635, 95% CI = 0.582–0.688) **(B)**. **(C, D)** DCA of the nomogram for the 1-, 3-, and 5-year survival prediction of patients in the training group (C-index = 0.594, 95% CI = 0.566–0.622) **(C)** and those in the validation group (C-index = 0.635, 95% CI = 0.582–0.688) **(D)**.

To increase the predictive prognostic value of TMB, we established a prognostic model for CRC patients with *KRAS* mutations. A total of 674 cases with complete clinical information were included in this analysis, of which 485 were randomly assigned to the training group and 189 to the validation group (**Table 5**). There was no significant difference between the training and validation cohorts for any of the included variables. We established a predictive nomogram model based on the clinical characteristics of the patients in the training group. The total scores for CRC patients with *KRAS* mutations can be computed to estimate the 1-, 3-, and 5-year survival rates, which would assist clinicians in assessing the risk of these patients in clinical practice (**Figure 3**). The survival model was assessed using the C-index and calibration plots. The C-index of the nomogram was 0.594 (95% CI = 0.566–0.622), and the calibration curve showed high consistency in the predicted outcomes of the survival model in both the training and validation cohorts (**Figures 4A, B** **)**. The results of the DCA showed that the nomogram had good accuracy in predicting the OS of CRC patients with *KRAS* mutations (**Figures 4C, D** **)**.

**Table 5:** Comparison of the baseline characteristics between the training and validation groups.

Characteristics	Training group	Validation group	*p*-value
*N*	485	189	
Age, *n* (%)			0.768
<60	244 (50.3%)	92 (48.7%)	
≥60	241 (49.7%)	97 (51.3%)	
Sex, *n* (%)			0.736
Female	235 (48.5%)	95 (50.3%)	
Male	250 (51.5%)	94 (49.7%)	
TMB, *n* (%)			0.481
High	82 (16.9%)	37 (19.6%)	
Low	403 (83.1%)	152 (80.4%)	
Primary tumor location, *n* (%)			1.000
Left	279 (57.5%)	109 (57.7%)	
Right	206 (42.5%)	80 (42.3%)	
Stage at diagnosis, *n* (%)			0.149
I	32 (6.6%)	16 (8.5%)	
II	82 (16.9%)	42 (22.2%)	
III	121 (24.9%)	51 (27%)	
IV	250 (51.5%)	80 (42.3%)	

TMB, tumor mutation burden.

## Discussion

TMB has recently been used to predict the outcome of ICI therapy, acting as an emerging indicator in several cancers to differentiate patients with various malignancies who may derive benefit from immunotherapy (19, 20). TMB has also been reported to be a prognostic predictor in certain types of cancer, such as cutaneous melanoma (21) and bladder cancer (22). TMB can be used as an independent indicator to assess the likelihood of patients with CRC responding well to ICI treatment (23); however, the prognostic value of TMB in CRC remains unclear. Our study illustrated, for the first time, the role of TMB in predicting the survival likelihood of patients with CRC involving *KRAS* mutation. Firstly, the clinical features and gene mutation data of 1,655 patients with CRC were obtained from the MSKCC and TCGA databases. Of these, 1,376 patients with TMB ≤ 10 mut/Mb were classified into the TMB-low category, while 278 patients with TMB > 10 mut/Mb were assigned into the TMB-high category (one patient lacked TMB data). No significant difference in OS was observed between the included CRC patients in the TMB-high and TMB-low groups in this study. Therefore, a more in-depth analysis is required.

Mutations in driver genes have been shown to play a central role in cancer development (24), determining the malignant behavior of tumors and affecting patient survival. In this study, we investigated the gene mutation profiles of 1,655 patients with CRC from the MSKCC and TCGA cohorts. We found that *APC*, *KRAS*, *PIK3CA*, and *TP53* were the most common mutated genes in both cohorts. Furthermore, Kaplan–Meier analysis showed that CRC patients with *APC* mutations had longer OS than those with wild-type *APC*. In contrast, *KRAS* mutations were associated with poor prognosis. Previous studies have suggested that inactivating mutations or deletion of the *APC* tumor suppressor gene are an early event in the development of CRC (25). The detection of *APC* gene mutations is useful for the early diagnosis and personalized treatment of CRC. *KRAS* is a member of the *RAS* family of genes and is associated with human tumors. *KRAS* mutations are significantly associated with distant metastasis of CRC and indicate poor prognosis (26).

Several factors are known to influence the prognosis of patients, including psychosocial, socioeconomic, and clinical parameters, and these parameters are routinely used to optimize treatment outcomes, limit risks, and personalize therapeutic strategies (27). TNM assessment continues to be the gold standard for tumor classification (28). Recent studies have indicated that, in CRC, *KRAS* mutations are related to suppressed immune pathways and that patients with *KRAS* mutations have altered expression levels of several immune-related genes (29). However, the expression levels of these genes are not routinely assayed in clinical practice, which limits their prognostic application. Our findings indicate that, in patients with *KRAS* mutations, those in the TMB-high group had longer OS than those in the TMB-low group. Multivariate Cox regression analysis showed that TMB was an independent prognostic factor for CRC patients with *KRAS* mutations, but not for those with *APC* mutations.

*KRAS* mediates numerous signal transduction pathways and plays critical regulatory roles during cell proliferation. *KRAS* mutations lead to abnormal cell proliferation and oncogenic transformation, promote cancer metastasis, and increase the resistance of several cancer types, including CRC, to chemotherapy and epidermal growth factor receptor (EGFR)-targeted therapy (30). Recent studies have shown that *KRAS* mutations are associated with poor prognosis in CRC (31–33), which may be related to its accelerated metastatic characteristic (34) and its resistance to chemotherapy and cetuximab (35). *KRAS* mutations can interact with the IL-22 pathway and enhance tumor cell proliferation (36). Additionally, studies have shown that all *KRAS* codon 12 alterations and p.G13D mutations are associated with poor prognosis in patients with CRC (37). When combined with chemotherapy, bevacizumab is the most effective first-line treatment for CRC patients with metastasis and *KRAS* mutations (38, 39). Immunotherapy for patients with *KRAS* mutations has been reported in recent years, but no clear effects on the outcomes have been observed (40). However, *KRAS* mutations are associated with high microsatellite instability in CRC (41), suggesting sensitivity to ICI therapy (42). This, in turn, suggests that *KRAS*-mutant CRC is a unique type of cancer, differing in terms of the treatment scheme and prognosis. A high TMB indicates that the tumor expresses more neoantigens and a greater probability that the immune system will recognize the cancer cells. This may explain why patients with high TMB have better prognosis (43).

In patients with CRC undergoing surgical resection, the prognosis and management are based on the TNM classification. Although the T and N stages are still the main prognostic factors in the current analysis, prognostic factors unrelated to the TNM stage may also affect the prognosis of patients. A nomogram is an easy-to-use prognostic model that helps clinicians in evaluating the prognosis of patients and making clinical decisions. To improve the prognostic value of TMB in CRC patients with *KRAS* mutations, we divided the enrolled patients into the training and validation groups. A nomogram was constructed based on the clinical characteristics of the training group. The validation group was used to evaluate the accuracy of the nomogram. This study is the first to construct a prognostic model for CRC patients with *KRAS* mutations. Establishing a nomogram prognostic model greatly improves the clinical value of TMB. Moreover, it is useful for clinicians when evaluating the prognosis of CRC patients with *KRAS* mutations and making patient-tailored clinical decisions.

## Conclusion

In conclusion, *KRAS*-mutant CRC is associated with poor prognosis and requires special prognostic indicators. With the application of immunotherapy and targeted therapy, somatic mutations and TMB have become the commonly used data in the clinic, which makes TMB more valuable for prognostic prediction. In this study, we analyzed the somatic mutation and TMB data of patients with CRC and found that TMB was an independent prognostic factor in those with *KRAS* mutations. Our study does, however, have some limitations. This is a retrospective study using data of patients with CRC obtained from a publicly accessible database, which reduced the power of proof. Since the collection of large quantities of CRC case data is time- and resource-intensive, we first opted to use public data for retrospective analysis. Although the conclusions of this study have some limitations, our findings indicate that there is ample merit in collecting the data of patients with CRC from clinical studies to further verify the prognostic value of TMB in those with *KRAS* mutations.

## Data availability statement

The datasets presented in this study can be found in online repositories. The names of the repository/repositories and accession number(s) can be found below: TCGA cohort (https://portal.gdc.cancer.gov/) and MSKCC cohort (https://www.cbioportal.org/study/summary?id=crc_msk_2017).

## Author contributions

All authors listed have made a substantial, direct, and intellectual contribution to the work, and approved it for publication.

## Funding

This study was supported by the Shandong Natural Science Foundation (no. ZR2021MH339).

## Conflict of interest

The authors declare that the research was conducted in the absence of any commercial or financial relationships that could be construed as a potential conflict of interest.

## Publisher’s note

All claims expressed in this article are solely those of the authors and do not necessarily represent those of their affiliated organizations, or those of the publisher, the editors and the reviewers. Any product that may be evaluated in this article, or claim that may be made by its manufacturer, is not guaranteed or endorsed by the publisher.
